# List of Diseases from the 20th of November to the 20th of December, 1799; Being the Result of the Public and Private Practice of a Physician at the West End of the Town

**Published:** 1800-01

**Authors:** 


### STATE OF DISEASES IN LONDON.

*List of Diseases from the 20th of November to the 20th of December,* 1799; *being the result of the public and private Practice of a Physician at the West End of the Town.*

| | No. of Cases. |
|---|---|
| ACUTE DISEASES. | |
| Measles - - - - | 21 |
| Scarlatina Anginosa - | 8 |
| Hooping Cough - - | 5 |
| Small-pox - - - | 4 |
| Contagious malignant Fever | 9 |
| Catarrh - - - | 20 |
| Acute Rheumatism - | 6 |
| Inflammatory Sore Throat | 2 |
| Pneumonia - - - | 3 |
| Peritoneal Inflammation - | 2 |
| Phlegmone Testis - - | 1 |
| Shingles - - - | 1 |
| Hectic and Slow Fever - | 8 |
| Childbed and Milk Fevers | 3 |
| Acute Diseases of Infants | 8 |
| CHRONIC DISEASES. | |
| Cough and Dispnœa - | 37 |
| Hæmoptoe and Phthisis - | 10 |
| Chronic Rheumatism - | 16 |
| Lumbago and Sciatica - | 5 |
| Asthenia - - - | 16 |
| Dropsy - - - | 6 |
| Cephalæa and Vertigo - | 10 |
| Paralysis - - - | 2 |
| Epilepsy - - | 3 |
| Hysteria - - - | 1 |
| Lethargy - - - | 1 |
| Dyspepsia - - | 15 |
| Gastrodynia - - - | 8 |
| Enterodynia - - | 6 |
| Jaundice - - - | 1 |
| Diarrhœa - - - | 8 |
| Colica Pictonum - - | 2 |
| Hæmorrhoids - - | 2 |
| Worms - - - | 3 |
| Amenorrhea - - | 4 |
| Menorrhagia - - | 1 |
| Abortus - - - | 2 |
| Fluor Albus - - | 1 |
| Hæmaturia - - - | 2 |
| Gravel and Dysury - | 3 |
| Scrophula - - - | 3 |
| Cancer - - - | 1 |
| Dentition and Tooth-Rash | 4 |

Itch

| | | | |
|---|---|---|---|
| Itch and Prurigo | 6 | Grocer's Itch | 5 |
| Lepra | 4 | Porrigo | 3 |
| Icthyoſis | 1 | Acne | 2 |
| Urticaria | 1 | Elephantiaſis | 1 |

No unfavourable caſe of meaſles has yet occurred. The ſcarlatina, however, has been attended with violent ſymptoms, and proved in two inſtances fatal. It is worthy of remark, that when the ſcarlatina, or contagious malignant fevers, are fully formed, and a froſt takes place ſuddenly, both diſeaſes are thereby much aggravated, and often terminate fatally; but, at the ſame time, that the farther diffuſion of theſe and other epidemical contagious diſorders is uſually prevented by a very cold atmoſphere.

Since the commencement of the preſent froſt, few malignant fevers have appeared: but this advantage may not be perhaps a ſubject of congratulation, as the pulmonic diſeaſes which ariſe during a ſevere winter, are uſually much more extenſive and more deſtructive than fevers.

*Red Lion Square, Dec.* 21, 1799. R. W.

